# Meal Time Shift Disturbs Circadian Rhythmicity along with Metabolic and Behavioral Alterations in Mice

**DOI:** 10.1371/journal.pone.0044053

**Published:** 2012-08-27

**Authors:** Ji-Ae Yoon, Dong-Hee Han, Jong-Yun Noh, Mi-Hee Kim, Gi Hoon Son, Kyungjin Kim, Chang-Ju Kim, Youngmi Kim Pak, Sehyung Cho

**Affiliations:** 1 Department of Neuroscience and Neurodegeneration Control Research Center, Kyung Hee University, Seoul, Korea; 2 Department of Legal Medicine, Korea University College of Medicine, Seoul, Korea; 3 Department of Biological Sciences, Seoul National University, Seoul, Korea; 4 Department of Physiology, Kyung Hee University School of Medicine, Seoul, Korea; Pennsylvania State University, United States of America

## Abstract

In modern society, growing numbers of people are engaged in various forms of shift works or trans-meridian travels. Such circadian misalignment is known to disturb endogenous diurnal rhythms, which may lead to harmful physiological consequences including metabolic syndrome, obesity, cancer, cardiovascular disorders, and gastric disorders as well as other physical and mental disorders. However, the precise mechanism(s) underlying these changes are yet unclear. The present work, therefore examined the effects of 6 h advance or delay of usual meal time on diurnal rhythmicities in home cage activity (HCA), body temperature (BT), blood metabolic markers, glucose homeostasis, and expression of genes that are involved in cholesterol homeostasis by feeding young adult male mice in a time-restrictive manner. Delay of meal time caused locomotive hyperactivity in a significant portion (42%) of subjects, while 6 h advance caused a torpor-like symptom during the late scotophase. Accordingly, daily rhythms of blood glucose and triglyceride were differentially affected by time-restrictive feeding regimen with concurrent metabolic alterations. Along with these physiological changes, time-restrictive feeding also influenced the circadian expression patterns of low density lipoprotein receptor (LDLR) as well as most LDLR regulatory factors. Strikingly, chronic advance of meal time induced insulin resistance, while chronic delay significantly elevated blood glucose levels. Taken together, our findings indicate that persistent shifts in usual meal time impact the diurnal rhythms of carbohydrate and lipid metabolisms in addition to HCA and BT, thereby posing critical implications for the health and diseases of shift workers.

## Introduction

Life on this rotating planet is confronted with periodic changes in environmental conditions. Yet, some of the environmental changes, such as environmental illumination, temperature, food/predator availability, are quite predictable throughout a day/night cycle. Thus, virtually all organisms on Earth have successfully evolved endogenous mechanisms that allow organisms to harmonize their behavioral and physiological processes according to time of day. The resulting circadian rhythms are believed to optimize energy utilization, reproduction and survival [1], [2].

The importance of endogenous circadian rhythms becomes evident when they are disrupted. For example, genetic defects within the core clock genes or circadian disturbances has been linked to various pathologies including obesity, metabolic syndrome, cancer, cardiovascular diseases, gastric disorders, and other physical and mental problems [3]–[5].

Humans have evolved to be active predominately during the light phase [6], [7]. In modern society, however, more and more workers are involved in various kinds of shift works. Shift work forces people to be active in a phase of the light-dark cycle during which normally they would be resting. Epidemiological evidence also indicates that shift works are closely associated with increased risk for obesity, type 2 diabetes, cardio-metabolic consequences, and sleep disturbances [5]. Yet, the precise mechanism(s) underlying these pathological changes needs to be resolved. To this end, however, proper animal models are necessary to delineate the specific aspects of circadian disruptions.

Some investigators have already utilized animal models for ‘shift works (night work)’ [8]–[10]. However, these models have their own advantages and limitations, and fall short of mimicking important aspects of circadian disruption in human. Specifically, mimicking shifts in usual meal time under a given day/night cycle may reveal important aspects of circadian disturbances that lead to metabolic consequences, as the feeding/fasting cycle plays a crucial role in metabolic homeostasis. Actually, eating at unusual times of the day has been suspected to cause metabolic disturbances and gastrointestinal symptoms in shift workers [11]–[13].

Previously, it was demonstrated that one week of daytime feeding in nocturnal mice shifts the phases of core clock genes expression in the peripheral oscillators but not in the SCN central clock [14]. However, the physiological or pathological consequences of the uncoupling between central and peripheral clocks have yet to be determined.

Nocturnal mice are known to consume most of daily food intake during the early half of their scotophase [3]. So, an interesting question arises what would happen to their behavioral and metabolic rhythms when their usual meal time is significantly advanced or delayed. Also interesting will be whether chronic shifts in their usual meal time lead to any metabolic anomalies. In the present study, the effects of 6 h advance or 6 h delay of meal time was investigated by feeding young adult male mice in a time-restrictive manner. Using a computerized monitoring system, the effects of time-restrictive feeding on body temperature and home cage activity were continuously monitored. In addition, the effects on cholesterol and glucose homeostasis were also explored.

## Results

### Time-restrictive feeding dramatically but reversibly alters body temperature and home cage activity rhythms in young adult male mice

**Figure 1:**
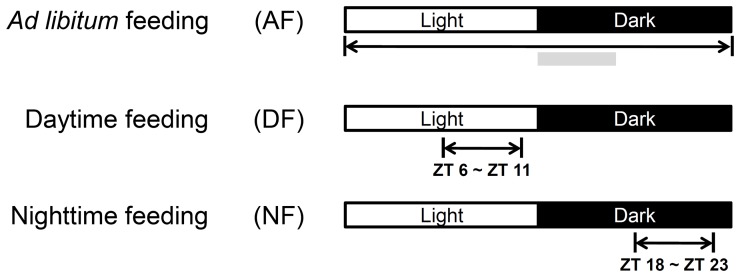
Experimental scheme. Young adult C57BL/6J male mice (8 weeks old) were first entrained to a 12∶12 LD photoperiodic cycle for two weeks with food and water available *ad libitum*. Then mice were randomly divided into three groups: food available *ad libitum* (AF), food available during the late day (ZT 6 to 11) (DF), and food available during the late night (ZT 18 to 23) (NF) with water available all the time. This time-restrictive feeding regimen was maintained either up to the end of each experiment or for 4 weeks and then returned to *ad libitum* feeding (Figure 2 and 3). Gray bar indicates major meal time of normal adult mouse (Figure S1A).

**Figure 2:**
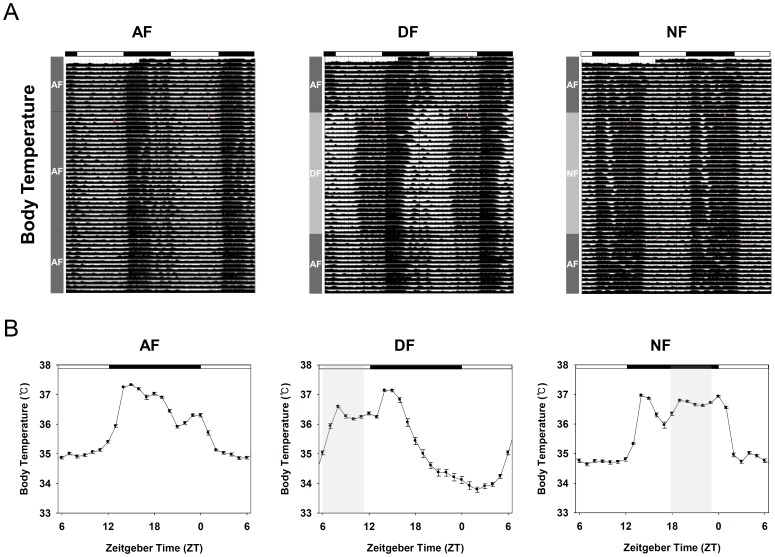
Daily rhythms of body temperature in young adult male mice under time-restrictive feeding regimen. Young adult male mice, surgically implanted with E-mitter probes, were first entrained to a 12∶12 LD photoperiodic cycle for two weeks with food and water available *ad libitum*. Then mice were fed time-restrictively as schematized in Figure 1 for 4 weeks and returned to being fed *ad libitum*. Body temperature (BT) was continuously recorded for 7 subsequent weeks (See *M&M*). (A) Representative double-plot actograms of BT in AF, DF, and NF mice. Dark rectangles above the actograms indicate the 12 h scotophase maintained throughout the experiment. (B) Daily patterns of BT during the time-restrictive feeding. To generate the daily pattern of BT, monitoring results for the whole time-restrictive period were averaged as 1 h bins and the resulting 28 day profiles were pooled according to the indicated ZT to generate the averaged daily pattern (mean ± S.E.M.). Statistical analyses are summarized in Table 1.

**Figure 3:**
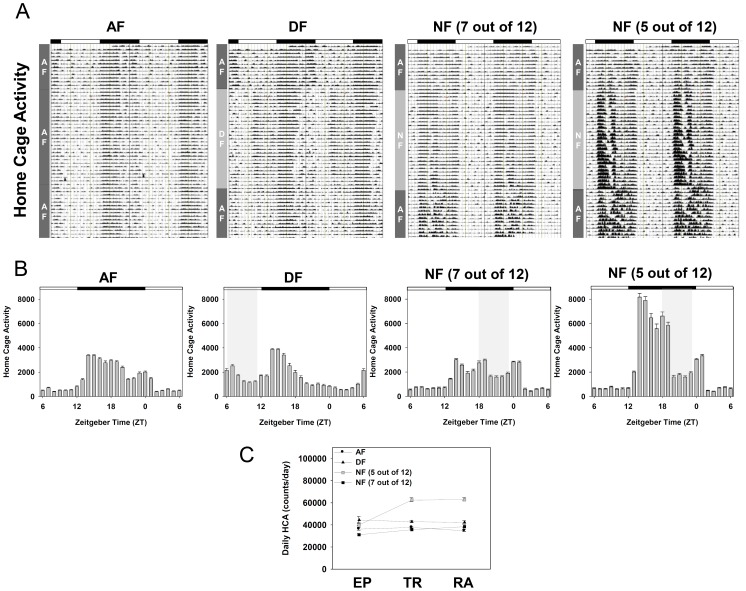
Daily rhythms of home cage activity in young adult male mice under time-restrictive feeding regimen. Young adult male mice, surgically implanted with E-mitter probes, were first entrained to a 12∶12 LD photoperiodic cycle for two weeks with food and water available *ad libitum*. Then mice were fed time-restrictively as schematized in Figure 1 for 4 weeks and returned to being fed *ad libitum*. Home cage activity (HCA) was continuously recorded for 7 subsequent weeks (See *M&M*). (A) Representative double-plot actograms of HCA in AF, DF, and NF mice. Dark rectangles above the actograms indicate the 12 h scotophase maintained throughout the experiment. (B) Daily patterns of HCA during the time-restrictive feeding. To generate the daily pattern of HCA, monitoring results for the whole time-restrictive period were summed up as 1 h bins and the resulting 28 day profiles were pooled according to the indicated ZT to generate the averaged daily pattern (mean ± S.E.M.). Statistical analyses are summarized in Table 1. (C) Changes in daily HCA during the weeks of entrainment period (EP), 4 weeks of time-restrictive feeding (TR) and when returned to *ad libitum* feeding for 2 weeks (RA).

As reported previously [3], adult C57BL/6J male mice consumed most of their food during the early scotophase when fed *ad libitum* **(Figure** **S1A)**. To see whether 6 h advance or 6 h delay of their usual meal time affects diurnal rhythms in physiology and metabolism, three experimental groups were adopted (**Figure 1**). Young adult male mice, surgically implanted with E-mitter probes, were entrained for two weeks to a 12∶12 LD photoperiodic cycle with food and water available *ad libitum*. Then mice were randomly divided into three groups. First group of mice (AF) were continuously fed *ad libitum* throughout the whole experimental period. The other two groups were fed either during the late day (DF group: ZT06∼ZT11) or during the late night (NF group: ZT18∼ZT23) for 4 weeks with water available all the time. Under the condition adopted, we confirmed that the amount of food intake in DF and NF groups was lower for the first 3∼4 days upon initiating time-restrictive feeding, but comparable amount of food were consumed within 4–5 days and thereafter as in AF group **(Fig S1B)**. After 4 weeks of time-restrictive feeding, all the mice returned to being fed *ad libitum*. Their body temperature (BT) and home cage activity (HCA) were continuously recorded at 6 min intervals, and their representative actograms are shown in **Figure 2A** **&** **3A**. As expected, distinct daily patterns of BT and HCA were evident in AF mice with rapid increase of BT and HCA at dusk, moderate dipping during the late night, and smaller but clear peak of BT and HCA at dawn (**Figure 2B** **&** **3B**). In contrast, both BT and HCA rhythms were dramatically altered by time-restrictive feeding regimens (**Figure 2** **&** **3**), and two-way ANOVA revealed that these altered daily rhythms of BT and HCA are the results of interactions between the feeding schedule and zeitgeber time (**Table 1**). Interestingly, rapid increases of BT and HCA at dusk were preserved regardless of feeding time, indicating that the peaking of BT and HCA at the start of scotophase is driven by the central clock set by the imposed LD cycle. However, this elevated BT and HCA in DF and NF mice were not sustained as much as in AF mice (**Figure** **2B** **&** **3B**), suggesting that the prolongation of elevated BT and HCA is dependent upon the food availability. Moreover, meal time clearly affected the shape of BT and HCA rhythms. In contrast to AF mice, food-anticipatory increases of BT and HCA were clearly seen in both DF and NF mice. In addition, the elevated BT during the meal time was sustained throughout the 5 h of feeding period while HCA was not much so. This suggests that the sustained increment of BT during the meal time is attributable to the food intake, but not to the increased activity. Another interesting point is that feeding during the late day caused torpor-like symptoms during the late night (**Figure 2B**, middle panel; see also **Figure S2** for individual profiles), indicating that a prolonged absence of food when it is most expected makes the animals enter energy-saving mode by lowering their BT. Importantly, the changes in BT and HCA rhythms induced by time-restrictive feeding seemed quite reversible as normal BT and HCA rhythms were recovered within a week when they returned to being fed *ad libitum* (**Figure 2A** **&** **3A**). Intriguingly, 6 h delay of usual meal time caused hyperactivity in some animals. Of 12 NF animals recorded, five mice showed increased daily activity during the restrictive feeding period (**Figure 3**). Moreover, the NF-induced hyperactivity was sustained even when they were fed *ad libitum* again (**Figure 3C**).

**Table 1:** ANOVA *F* and *p* values for BT and HCA rhythms.

Physiological indices	Factors		
	Feeding Schedule	Zeitgeber Time	Interaction
Body Temperature	***F*** (2,1944) = 364.85; ^****^ ***p*** **<0.0001**	***F*** (23,1944) = 363.98; ^****^ ***p*** ** <0.0001**	***F*** (46,1944) = 150.34; ^****^ ***p*** ** <0.0001**
Home Cage Activity	***F*** (2,1944) = 381.29; ^****^ ***p*** ** <0.0001**	***F*** (23,1944) = 420.83; ^****^ ***p*** ** <0.0001**	***F*** (46,1944) = 71.36; ^****^ ***p*** ** <0.0001**

Significant differences (****p<0.0001) are indicated in bold type.

ANOVA, analysis of variance.

### Food and water intakes are marginally affected by time-restrictive feeding regimen

**Figure 4:**
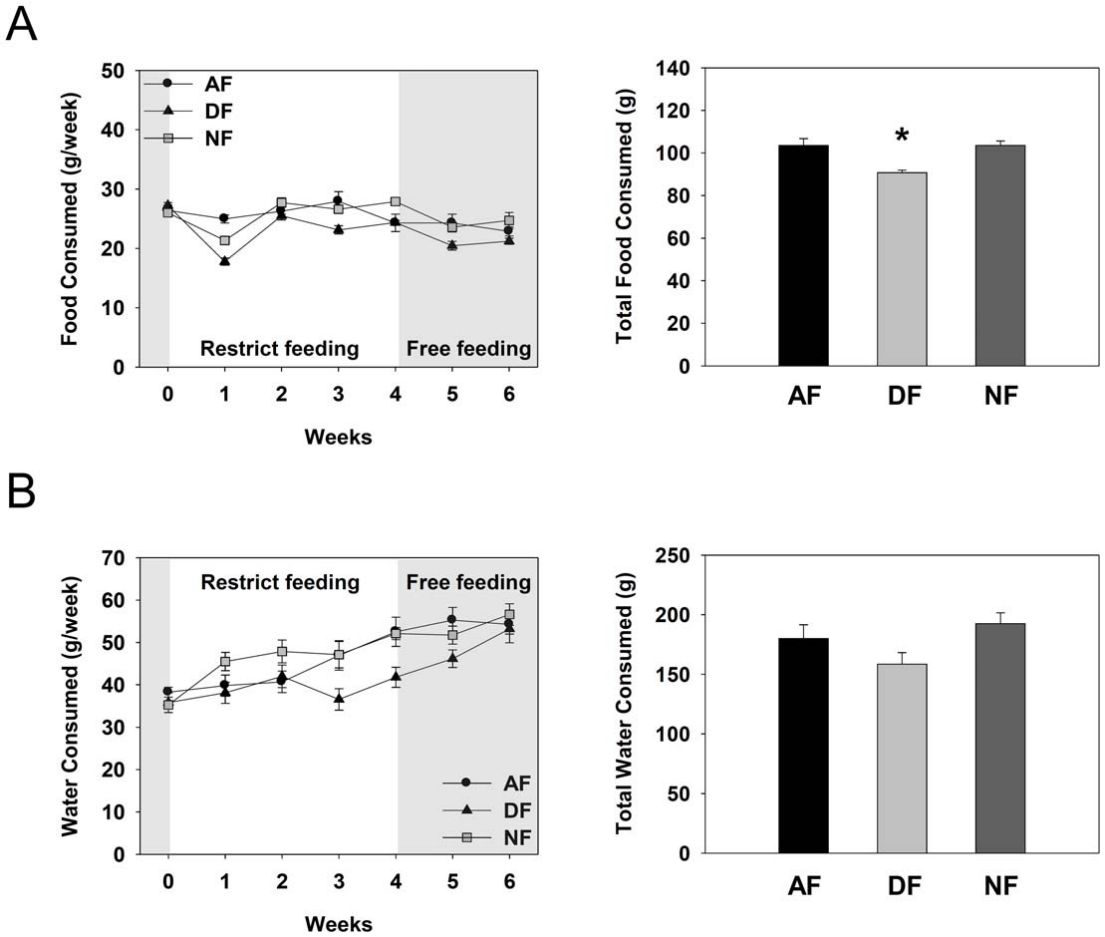
Weekly food and water consumption profiles. Young adult male mice were first entrained to a 12∶12 LD photoperiodic cycle for 1 week, fed time-restrictively for 4 weeks, and then fed *ad libitum* for 2 weeks as described in Figure 1. Weekly consumption of food and water measured regularly at the end of each week. (A) Weekly food consumption profiles during the whole experimental period (left) and total food consumption during the 4 weeks of time restrictive feeding period (right). (B) Weekly water consumption profiles during the whole experimental period (left) and total water consumption during the 4 weeks of time restrictive feeding period (right). Data are expressed as mean ± S.E.M. (n = 4), *p<0.05 *vs*. other groups.

To examine whether time-restrictive feeding affects the amount of food and water intakes, weekly food and water consumptions were measured throughout the whole experimental period. As shown in **Figure 4A**, food intakes were significantly lower during the first week of restrictive feeding, especially in DF mice, but comparable food consumption was recovered by the second week and thereafter. Total food consumption during the 4 weeks of time-restrictive feeding was marginally but significantly lower in DF group than in other groups (**Figure 4A**, right panel). Weekly water intakes were also reduced by daytime feeding regimen especially at the third and fourth weeks of restrictive feeding, which were progressively recovered by returning to *ad libitum* feeding (**Figure 4B**, left panel). Yet, the total water intakes during the 4 weeks of time-restrictive feeding were statistically indistinguishable among the three groups (**Figure 4B**, right panel). Thus, it can be concluded that nighttime feeding does not affect weekly food and water intakes while daytime feeding marginally reduces the weekly food and water consumption.

### One week of time-restrictive feeding dramatically alters the daily rhythms of metabolic parameters

**Figure 5:**
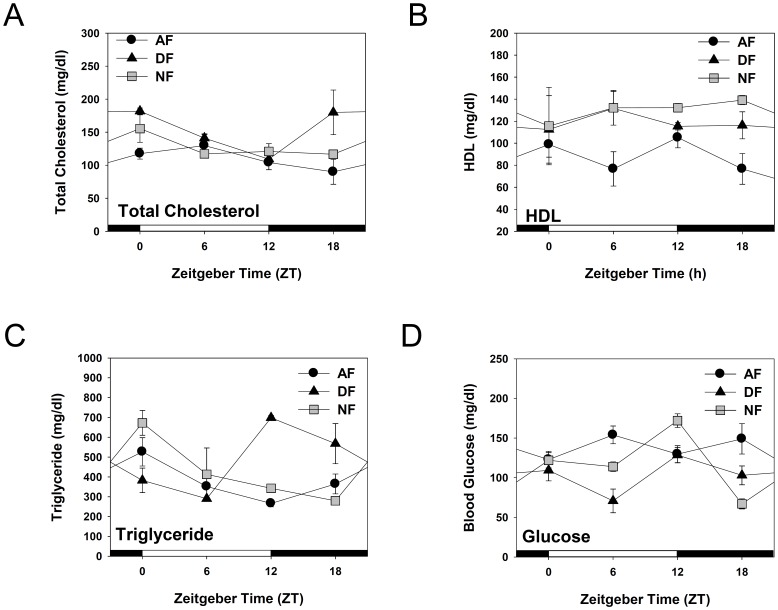
Daily rhythms of blood glucose and some metabolic parameters related to cholesterol homeostasis in mice fed time-restrictively. Young adult male C57BL/6J mice were first entrained to a 12∶12 LD photoperiodic cycle for two weeks. Then mice were fed time-restrictively for seven consecutive days as denoted in Figure 1. Mice were sacrificed by cervical dislocation at the indicated ZT and whole blood samples were collected. Total cholesterol (A), HDL cholesterol (B), plasma triglyceride (C), blood glucose (D) levels were determined by specific kits obtained from Callegari^TM^. All data are expressed as mean ± S.E.M. (n = 4–8). Statistical analyses are summarized in Table 2.

Recent studies indicate that circadian disruption may potentiate the onset of metabolic disorders. For example, genetic defects within the core clock genes *Clock* and *Bmal1* have been associated with obesity, metabolic phenotypes, hypertension, and type 2 diabetes [15]–[17]. To test whether the altered BT and HCA rhythms induced by time-restrictive feeding regimen are also associated with disturbances in metabolic rhythms, daily variation in the levels of total cholesterol, high-density lipoprotein (HDL) cholesterol, triglyceride, and blood glucose were determined after a week of time-restrictive feeding (**Figure 5**). When nocturnal mice were fed *ad libitum*, total cholesterol levels remained fairly stable throughout a circadian cycle but slightly decreased at the time of light-dark transition. HDL cholesterol remained rather stable throughout a day. Blood triglyceride levels seemed to be mainly affected by the meal time, since they reached the highest level after the usual meal time and progressively declined thereafter. Blood glucose levels were fairly stable when mice were fed *ad libitum*. When mice were fed time-restrictively, however, these metabolic rhythms were differentially affected. In case of total cholesterol and HDL, the effects were rather moderate (**Figure 5A&B**). Once again, blood triglyceride levels seemed to be mainly affected by the meal time. As shown in **Figure 5C**, blood triglyceride levels reached the maximum after the meal time (ZT12 in DF and ZT00 in NF mice) and progressively declined thereafter. On the other hand, blood glucose reached the minimum before the meal time (ZT6 in DF and ZT18 in NF mice) and elevated after the foraging (**Figure 5D** **)**. Also notable is the sharp increase of blood glucose in NF mice at the light/dark transition. When combined with the activity onset observed in these mice (**Figure 2** **&** **3**), it is probable that this rise in blood glucose may reflect the metabolic needs required for the activity onset. Two-way ANOVA revealed that these altered daily rhythms of total cholesterol, triglyceride, and glucose are the results of interactions between the feeding schedule and zeitgeber time (**Table 2**). Thus, we conclude that just a week of time-restrictive feeding differentially affects the metabolic rhythms, thereby destroying the harmony among the metabolic parameters.

**Table 2:** ANOVA *F* and *p* values for each physiological index.

Physiological indices	Factors			
	Feeding Schedule	Zeitgeber Time	Interaction	
Total Cholesterol	***F*** (2,44) = 8.84; ^***^ ***p*** ** = 0.0006**	***F*** (3,44) = 4.47; ^**^ ***p*** ** = 0.0080**	***F*** (6,44) = 2.45; ^*^ ***p*** ** = 0.0393**	
HDL	***F*** (2,43) = 6.46; ^**^ ***p*** ** = 0.0035**	*F*(3,43) = 0.15; ^ns^ *p* = 0.9259	*F*(6,43) = 0.62; ^ns^ *p* = 0.7125	
Triglyceride	*F*(2,43) = 3.03; ^ns^ *p* = 0.0589	***F*** (3,43) = 3.21; ^*^ ***p*** ** = 0.0324**	***F*** (6,43) = 6.55; ^****^ ***p*** ** <0.0001**	
Blood glucose	***F*** (2,48) = 8.23; ^***^ ***p*** ** = 0.0008**	***F*** (3,48) = 4.83; ^**^ ***p*** ** = 0.0051**	***F*** (6,48) = 5.75;^***^ ***p*** ** = 0.0001**	

Significant differences (*p<0.05, **P<0.01, ***P<0.001 and ****p<0.0001) are indicated in bold type.

ANOVA, analysis of variance; ns, not significant.

### LDLR, SREBP1C and SCAP mRNAs oscillate in the mouse liver

**Figure 6:**
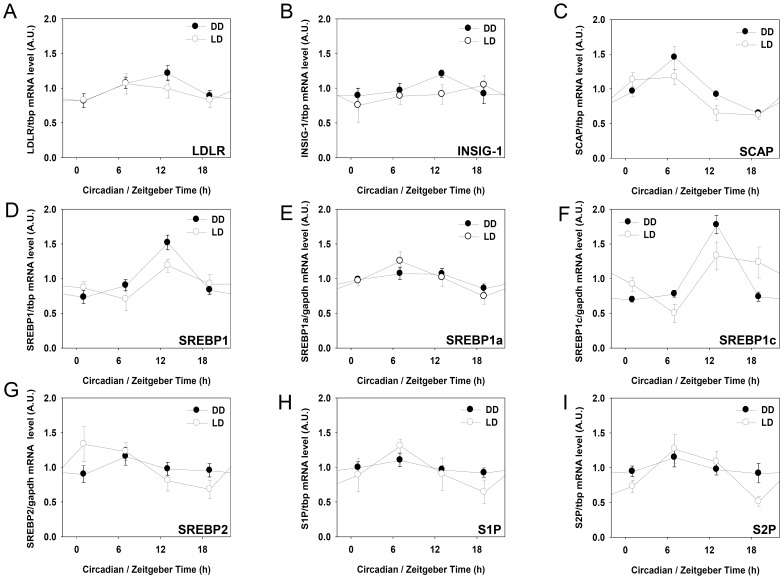
Daily and circadian expressions of LDLR and some LDLR regulatory factors in the mouse liver. To determine daily expression patterns, young adult C57BL/6J male mice were entrained to a 12L:12D cycle for two weeks and liver samples were quickly obtained at the indicated ZT. For circadian sampling, mice were entrained to a 12L:12D cycle for two weeks and released to constant darkness (DD). On the second day after light-off, liver samples were obtained at the indicated circadian time (CT). RNA isolation, reverse transcription, and real-time polymerase chain reaction to measure specific messages for mouse *ldlr*, and LDLR regulatory factors. All mRNA levels were normalized to *tbp* mRNA levels. Data are expressed as mean ± S.E.M. (n = 8).

Daily variation of metabolic parameters related to cholesterol metabolism was altered by time-restrictive feeding (**Figure 5A-C**). Cholesterol metabolism is controlled by various factors including low density lipoprotein receptor (LDLR), sterol regulatory element-binding protein-1 (SREBP-1), sterol regulatory element-binding protein-2 (SREBP-2), SREBP-cleavage activating protein (SCAP), insulin-induced gene-1 (INSIG-1), site-1 protease (S1P), and site-1 protease (S2P) [18]–[20]. Previously, LDLR has been shown to have a circadian rhythm in the rat [21]. To examine whether the LDLR and LDLR regulatory factors expressions exhibit daily and/or circadian rhythms in the mouse liver, young adult male mice were first entrained to a 12∶12 LD photoperiodic cycle for two weeks. Then, mice liver samples were obtained throughout a day in the presence of LD cycle, or throughout a circadian cycle under DD condition on the second day after light-off. As shown in **Figure** **6**, *ldlr* and some LDLR regulatory factors expression oscillated in the mouse liver in the presence or absence of exogenous light cues. Notably, the *ldlr, srebp1* and *srebp1c* mRNA levels peaked at ZT13 or CT13, while *scap* mRNA levels reached the maximum at ZT07 and CT07. The *insig*, *srebp-1a*, *srebp2*, *s1p*, and *s2p* mRNA levels did not exhibit any significant circadian variations.

### One week of time-restrictive feeding dramatically alters the daily expression of LDLR and LDLR regulatory factors

**Figure 7:**
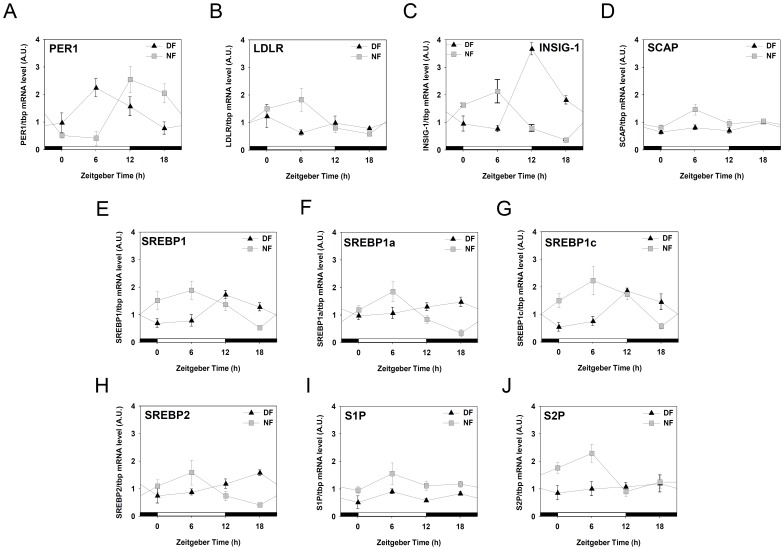
Effects of one week of time-restrictive feeding on the phases of Per1, LDLR, and LDLR regulatory factors gene expression in the mouse liver. Young adult male mice, entrained to a 12∶12 photoperiodic cycle, were fed time-restrictively for seven consecutive days as described in Figure 1. On the 8^th^ day, mice were sacrificed at the indicated zeitgeber time (ZT) and liver samples were obtained. RNA isolation, reverse transcription, and real-time polymerase chain reaction were performed to measure specific messages for mouse *Per1*, *ldlr*, and LDLR regulatory factors. All mRNA levels were normalized to *tbp* mRNA levels. Data are expressed as mean ± S.E.M. (n = 4).

Previously, it has been shown that one week of daytime feeding shifts the phase of circadian genes expression in the peripheral oscillators like liver [11], [22]. With the circadian oscillation of LDLR and some LDLR regulatory factors expression in hands (**Figure 6**), next question was whether these oscillations would be affected by time-restrictive feeding regimen. Young adult male mice were entrained to a 12∶12 photoperiodic cycle for two weeks and fed either during the late day (DF group: ZT06∼ZT11) or during the late night (NF group: ZT18∼ZT23) for 7 consecutive days with water available all the time. On the eighth day, mice were sacrificed at 6 h intervals, liver samples were obtained, and messages for the *ldlr* and LDLR regulatory factors were analyzed using real-time PCR. As shown in **Figure 7A**, the expression of a core clock gene, *per1,* clearly phase-shifted by time restrictive feeding regimen. Moreover, the phases of the *ldlr* and most LDLR regulatory factors expression were significantly altered by time-restrictive feeding regimen. Specifically, all mRNA except *s1p* reached the maximum at ZT06 in NF mice, while *insig-1*, *scap*, *srebp1*, *srebp1a*, *srebp1c*, and *srebp2* reached the highest level at ZT 12–18 in DF mice. Interestingly, the phases of all mRNA accumulation profiles differed by 6–12 h between DF and NF mice. These results indicate that the daily expression of LDLR and LDLR regulatory factors are severely affected by time-restrictive feeding regimen in the mouse liver.

### Chronic time-restrictive feeding significantly affects glucose homeostasis in young adult male mice

**Figure 8:**
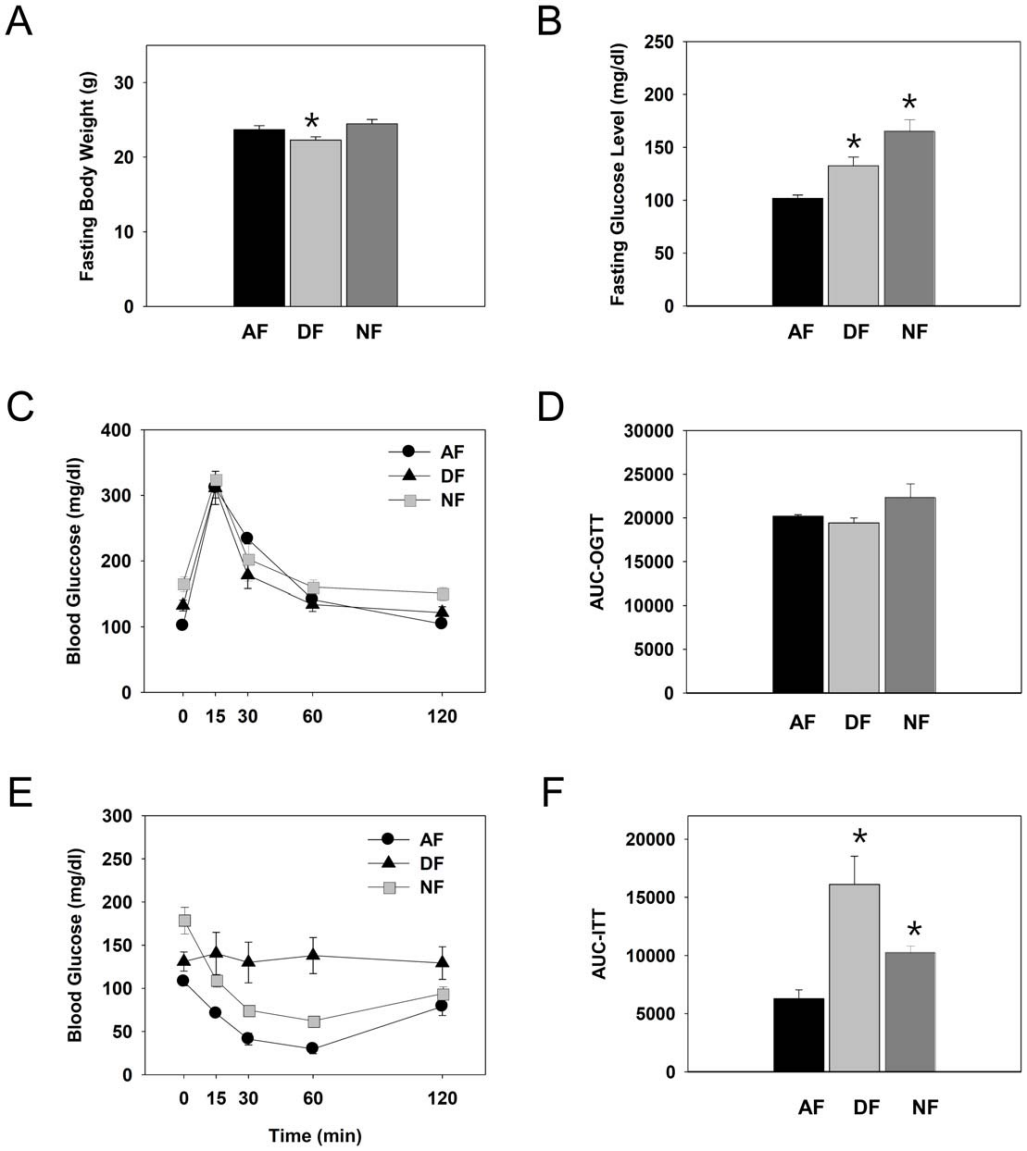
Effects of chronic time-restrictive feeding on body weight, fasting blood glucose, glucose tolerance, and response to insulin. Young adult male mice were first entrained to a 12∶12 LD photoperiodic cycle for 2 weeks and then fed time-restrictively for 9 consecutive weeks. After 16 h fasting, mice were weighed (A) and fasting glucose levels were measured (B) at the 5^th^ week. Then oral glucose tolerance test was performed. Blood glucose levels over time in response to an oral glucose load (C) and area under the curve for OGTT (D) are shown. At the 9^th^ week, mice were weighed after 16 h fasting, and fasting glucose levels were measured. Then insulin tolerance test was performed. Blood glucose levels over time in response to insulin (E) and area under the curve (F) were determined. All data are expressed as mean ± S.E.M. (n = 3–4), *p<0.05 *vs*. control AF group.

Since daily patterns of blood glucose levels were significantly altered by time-restrictive feeding regimen (**Figure 5D**), it was tempting to examine whether these changes lead to any metabolic consequences. Thus, young adult male mice were first entrained to a 12∶12 LD photoperiodic cycle for two weeks. Then, mice were fed time-restrictively for 9 consecutive weeks. Changes in body weight, fasting blood glucose, tolerance to the oral glucose load, and response to the insulin challenge were determined weekly from the 5^th^ week of restrictive feeding and thereafter. As shown in **Figure 8A**, fasting body weights were slightly but significantly reduced in DF mice compared to other groups, probably reflecting the reduced food intake in these mice (**Figure 4A**). More significant impacts were observed in fasting glucose levels. As shown in **Figure 8B**, fasting glucose levels were significantly higher in mice fed time-restrictively, especially in NF mice. These elevations of fasting glucose levels were observed from the 5^th^ week of time-restrictive feeding to the end of experiment at the 9^th^ week. However, the responses to the oral glucose load were almost indistinguishable among the three groups, not only at the 5^th^ week (**Figure 8C&D**) but to the end of experiment (data not shown). Importantly, responses to the insulin challenge exacerbated as the daytime feeding regimen continued. Specifically, altered responses to insulin were already notable from the 6^th^ week of time-restrictive feeding (data not shown) and insulin resistances were evident at the 9^th^ week (**Figure 8E&F**). Thus, it can be concluded that homeostatic regulation of blood glucose are significantly and progressively affected by shift in usual meal time.

## Discussion

The present study explored the effects of 6 h advance or 6 h delay of usual meal time in young adult male mice, and found that both BT and HCA rhythms were significantly altered by shifts in meal time with concurrent pathological changes in metabolic rhythms. Since shift workers are known to be exposed to high risks of metabolic problems [23], the observation that time-restrictive feeding alters daily variation of factors regulating glucose and cholesterol homeostasis poses great implications that necessitate future works.

Previously, Damiola et al. [14] observed that daytime-restrictive feeding alters the phases of core clock genes expression in the peripheral tissues, but not in the central clock SCN. In the present study, rapid elevations of both BT and HCA at the early scotophase were preserved regardless of feeding regimens, indicating that these increases are mainly controlled by the central clock set by the imposed LD cycle. Accordingly, *mPer1* ^−/−^ *mCry2* ^−/−^ clock mutant mice did not exhibit these peaks [24], [25], supporting the dominant role of central clock machinery in the activity onset. Nevertheless, the prolongation of elevated BT and HCA seems to be dependent upon food availability, because they were not sustained as much in the absence of food. Moreover, food availability significantly affected the BT and HCA rhythms. Interestingly, a prolonged absence of food when it is most expected induced the regulated hypothermia in DF animals (**Figure 2B** and individual profiles in **Figure S2B**). Torpor has been believed to be an energy-saving mode that enables the small animals to endure prolonged food-deficient period [26], [27]. Yet, torpor-like symptom was observed only in DF mice, but not in NF mice with the same 19 h fasting period a day, suggesting an unexpected role of circadian clock in the physiological regulation of torpor-like symptoms. Leptin and ghrelin, which are known to affect torpor [28], [29], exhibit daily variations in rodents [30]. Recently, Crispim et al. [31] observed that leptin and ghrelin levels showed significant differences from the day shift subjects in human. Thus, time-restrictive feeding may alter daily patterns of leptin and ghrelin, leading to a torpor-like symptom. This possibility needs to be addressed in the future works.

**Table 3:** Primer sequences used in real-time PCR.

Gene		Sequence	NCBI ID
LDLR	Forward	5′-GAA CTC AGG GCC TCT GTC TG-3′	NM_010700
	Reverse	5′-AGC AGG CTG GAT GTC TCT GT-3′	
SREBP1	Forward	5′-GTG AGC CTG ACA AGC AAT CA-3′	NM_011480
	Reverse	5′-GGT GCC TAC AGA GCA AGA GG-3′	
SREBP1a	Forward	5′-CGG CTC TGG AAC AGA CAC TG-3′	NM_011480
	Reverse	5′-AAG TCA CTG TCT TGG TTG TTG-3′	
SREBP1c	Forward	5′-ATC GGC GCG GAA GCT GTC GG-3′	NM_011480
	Reverse	5′-AAG TCA CTG TCT TGG TTG TTG-3′	
SREBP2	Forward	5′-GTG GAG CAG TCT CAA CGT CA-3′	NM_033218
	Reverse	5′-TGG TAG GTC TCA CCC AGG AG-3′	
SCAP	Forward	5′-GAT GTG TTC CGG TCA CCT CT-3′	NM_001103162
	Reverse	5′-TTG GTC CCT GAG CTGTCT CT-3′	
INSIG-1	Forward	5′-ACA CGTGGG ACC TAA CTT GC-3′	NM_153526
	Reverse	5′-CTT CTC CGG AAT AGC TCG TG-3′	
S1P	Forward	5′-TCC CCA GCA GAG ACA GAG TT-3′	NM_019709
	Reverse	5′-GTG GTA CTG GTC CCA GAG GA-3′	
S2P	Forward	5′-CAC TGG GAC TCT GGA TGG TT-3′	NM_172307
	Reverse	5′-TTG GCC AAG GTG TTT TAA GG-3′	
PER1	Forward	5′-GTG TCG TGA TTA AAT TAG TCA G-3′	NM_011065
	Reverse	5′-ACC ACT CAT GTC GTC TGG GCC-3′	

Another interesting point found in this work is that hyperactivity was observed in a significant portion of NF animals (42%, 5 out of 12 animals; **Figure 3A**, far right panel). Surprisingly, the NF-induced hyperactivity was continued even when they were fed *ad libitum* again, indicating a sustained aftereffect of 6 h delay of meal time on behavioral phenotypes. In this context, it is noteworthy that clinical evidence suggests a role of circadian time-keeping system in the etiology of attention-deficit/hyperactivity disorders (ADHD) [32]–[34]. Moreover, circadian rhythm disturbance has been linked to bipolar depression [35] one of whose symptom is hyperactivity [36]. Taken together, hyperactivity observed in NF animals can be associated with mental illnesses. Further works are needed to confirm this issue.

The present work demonstrated that chronic time-restrictive feeding induces differential metabolic changes along with the alteration in BT and HCA rhythms, which is in good agreement with the recent studies suggesting that circadian rhythm disturbances are linked to obesity, type 2 diabetes, and metabolic phenotypes [15], [17]. The present results showing the disturbed metabolic rhythms also correspond with previous reports in rodents and human studies that eating during the normal rest phase lead to a loss of blood glucose and triglyceride rhythms [10], [37]–[39].

Cholesterol is an important source of bile acid, biological membrane, and steroid hormones [18], and increased plasma cholesterol has been associated with an increased risk of coronary heart disease and atherosclerosis [40]. LDLR is known to play a key role in cholesterol metabolism. The transcription of the LDLR is regulated by SREBP pathway. SREBP pathway is composed of various factors including SREBP-1a, SREBP-1c, SREBP-2, INSIG-1, SCAP, S1P, and S2P [19], [20], [41], [42]. The present study examined whether mRNA expressions of these factors oscillate in the mouse liver and found that LDLR, SREBP1, and SREBP1c expressions oscillated in the mouse liver under free-running condition that is in good agreement with previous reports [21], [43]. Moreover, it was also found that *scap* mRNA oscillates with maximum peak at CT07 (**Figure 6C**), suggesting that LDLR and some LDLR regulatory factors are clock-controlled genes.

More importantly, the present study revealed that just a week of meal time shift disturbed the daily patterns of LDLR and most of LDLR regulatory factors expressions (**Figure 7**). Severe impacts of time-restrictive feeding on the daily expression of genes regulating cholesterol homeostasis may have critical implications for shift workers, since they are known to be more vulnerable to cardiovascular diseases [44]–[46]. However, chronic effects of time-restrictive feeding on cholesterol homeostasis and related pathologies need to be addressed by the future works. In this respect, a recent observation that interaction between temporal feeding and circadian clock determines the hepatic circadian transcriptome [22] provides a valuable clue. Future works addressing which genes are driven by feeding only, circadian clock only, or interaction of both would help understand the pathophysiology related to chronic shift of meal time.

Blood glucose levels are controlled by many factors such as leptin, insulin, and corticosterone [47], [48]. In particular, altered responses to insulin or to glucose load indicate disturbances in glucose homeostasis [49]. Since daily variations in circulating glucose levels were significantly influenced by time-restrictive feeding regimens (**Figure 5D**), the present work implies that metabolic syndrome can ensue with significant shifts in meal time. Yet, the direction of shifts seemed to make a difference. In the present study, fasting glucose levels were most severely affected by 6 h delay in meal time (NF mice; **Figure 8**), while altered response to insulin were observed only by 6 h advance (DF mice; **Figure 8E&F**) without altering the serum insulin levels (data not shown). At present, it is unclear what made such differences. One possibility is that, as mice are born to be predominantly active during the scotophase, 6 h advance of meal time makes them active during their usual rest phase, imposing more severe stress in these animals, while 6 h delay causes prolonged activities during the night with increased metabolic needs. Alternatively, the direction of shifts causes differential influences on the hormonal secretion and insulin signaling. As insulin signaling pathways involve insulin receptor, phosphatidylinostiol-3kinase (PI3K), phosphatidylinositol-4,5-bisphosphte (PIP_2_), and PIP_3_-dependent protein kinase (PDK1) [**50**], [**51**] and virtually all hormones display diurnal variations [52], [53], future works are needed to address this issue.

In conclusion, the present study demonstrated that shifts in usual meal time greatly affects daily rhythms of HCA and BT, along with the alterations in daily rhythms of blood glucose and triglyceride levels, leading to some metabolic consequences. These data pose critical implications for the health and diseases of shift workers that are ever increasing in the modern society.

## Materials and Methods

### Ethics statement

All animal experiments were approved by the Kyung Hee University Institutional Animal Care and Use Committee (Permit number: KHUASP(SE)-11-035) and performed under the guidelines of the Committee. All the animals were treated to minimize suffering.

### Animals care and handling

C57BL/6J male mice were purchased from DBL (Seoul, Korea). Upon arrival, mice were acclimatized to a temperature-controlled room (23±1°C) with a 12∶12 light-dark (LD) photoperiodic cycle. Food and water were provided all the time. For experimental settings, mice were individually housed in a light-proof clean animal rack cabinet (Shin Biotech, Seoul, Korea) with light intensity during the light phase maintained at 350∼450 lux at the bottom of cage. Mice were continuously fed with normal food chows *ad libitum* until the start of time-restrictive feeding.

### Activity monitoring, restrictive feeding schedule and sampling

For activity monitoring, mice were surgically implanted with a G2 E-mitter probe (Mini Mitter, Oregon, USA) on the dorsal neck under the skin [54], [55], and their HCA and BT were continuously monitored using Activity Monitoring System (Mini Mitter, Oregon, USA). Specifically, data were continuously recorded at 6 min intervals using the VitalView^®^ Data Acquisition System. Individual actograms were obtained using the ActiView^®^ software. To generate the daily pattern, monitoring results retrieved as a Microsoft^®^ Excel file for the whole time-restrictive period were either averaged (in case of BT) or summed up (in case of HCA) as 1 h bins and the resulting 28 day profiles were pooled according to the indicated ZT. Time-restrictive feeding was applied two weeks after E-mitter implantation surgery. After a week of recovery, mice were further entrained for another week to a 12∶12 LD photoperiodic cycle with food and water available *ad libitum*. Then mice were randomly divided into three groups (**Figure 1**). First group of mice (AF) were continuously fed *ad libitum* throughout the whole experimental period. The other two groups were fed either during the late day (DF group: ZT6∼ZT11) or during the late night (NF group: ZT18∼ZT23) for weeks as indicated in the Figure legends. In case of Figure 2 and 3, all the mice returned to being fed *ad libitum* after 4 weeks of time-restrictive feeding. For circadian sampling (**Figure 6**), mice were first entrained for two weeks to a 12∶12 LD photoperiodic cycle with food and water available *ad libitum*. Then mice were released to constant darkness. On the second day after light-off, mice were sacrificed by cervical dislocation under the dim red light and liver samples were quickly obtained. To examine the effect of restrictive feeding on genes expression (**Figure 7**), mice were first entrained for two weeks to a 12∶12 LD photoperiodic cycle, fed either during the late day (DF group) or during the late night (NF group) for seven consecutive days, and liver samples were obtained at the indicated ZT.

### Measurement of metabolic parameters

Blood samples were collected from the retro-orbital plexus at the time of sacrifice. Blood glucose levels were determined using the Accu-Chek^®^ Performa kit (Roche, Seoul, Korea). After 30 min incubation at room termperature, blood samples were centrifuged at 3,000 rpm at 4°C for 10 min, and resulting serum was collected. Total cholesterol, HDL (high density lipoprotein) cholesterol, and triglyceride levels were determined using specific assay kits and CR-3000 apparatus obtained from Callegrari^TM^ (Parma, Italy).

### Oral glucose tolerance test (OGTT) and insulin tolerance test (ITT)

In a control experiment, we found that time-of-day has little impact on OGTT and ITT results (**Figure S3**). Thus, we performed OGTT and ITT after 16 h fasting since the last scheduled meal. For OGTT, D-glucose (Sigma, G5767; 2 g/kg body weight) was delivered using oral zonde. For ITT, insulin (0.5 U/kg body weight) was injected intraperitoneally after 16 h fasting. Blood samples were collected at 0, 15, 30, 60, and 120 min for determination of blood glucose levels.

### RNA isolation, reverse transcription, and real-time PCR

Total RNA was isolated from the mouse liver using single-step acid guanidinium thiocyanate-phenol-chloroform (AGPC) extraction method as described previously [56]. Concentration of RNA was determined using ND-1000 (Nanodrop Technologies, Wilmington, USA). RNA samples diluted to 1 μg /10 μl were incubated with 200 ng of random hexamer (Takara, 3801) at 65°C for 5 min, and rapidly cooled down on ice for 2 min. Then each sample was incubated with 9 μl of reverse transcription mixture (4 μl of RT buffer, 0.5 μl of RNase inhibitor, 4 μl of 2.5 mM ea of dNTPs and 0.5 μl of RTase M-MLV) at 37°C for 1 h and subsequently heated at 70°C for 10 min. The procedure for real-time PCR using LightCycler has been described [57]. The standards were prepared by pooling 5-fold diluted RT samples in 1 mM Tris. The templates for real-time PCR were prepared by diluting 75-fold from RT samples. 2X SYBR Premix EX Taq (Takara, RR041A) and LightCycler Version 1.5 (Roche, Salt Lake City, USA) were used for the real-time PCR. Primer sequences used for real-time PCR are shown in **Table 3**.

### Statistical analysis

Data were classified by group and time and were expressed as mean ± S.E.M. Statistical analyses were performed with GraphPad PRISM software (GraphPad Prism Software, Inc., LA Jolla, CA, USA). The factor analysis between groups was carried out using 2-way analysis of variance (ANOVA) with Bonferroni posttests. Food intake, water intake, fasting glucose, fasting body weight, AUC-OGTT, and AUC-ITT were statistically analyzed by ANOVA. Statistical significance was set at p<0.05.

## Supporting Information

Figure S1 (A) Hourly food intakes in mice fed *ad libitum*. Adult C57BL/6J male mice (n = 16) were entrained to a 12∶12 LD photoperiodic cycle for a week in metabolic cages with food and water available all the time. On the 8th day, hourly food intake was determined throughout a day. (B) Time-course changes of daily food consumption in mice fed at either ZT06-11 (DF) or ZT18-23 (NF). Average daily food consumption in *ad libitum* fed mice is shown as a dashed line within the figure as a reference value.
(TIF)

Figure S2 Representative 48 hour individual body temperature profiles in AF (A), DF (B) and NF (C) animals. Only in DF animals, rapid lowering of body temperature and recovering to normal ranges were repeated during the each fasting period.
(TIF)

Figure S3 Time-of-day has little effects on OGTT and ITT results. Adult C57BL/6J male mice were fasted from the indicated zeitgeber time (ZT) for 16 h. Then, OGTT (A) and ITT (B) were performed as described in *M&M* of the main text.
(TIF)
