# Supplementary figures and images for: Meal Time Shift Disturbs Circadian Rhythmicity along with Metabolic and Behavioral Alterations in Mice
Source: PLoS One. 2012 Aug 27;7(8):e44053. doi: 10.1371/journal.pone.0044053 (PMC3428308; doi:10.1371/journal.pone.0044053)

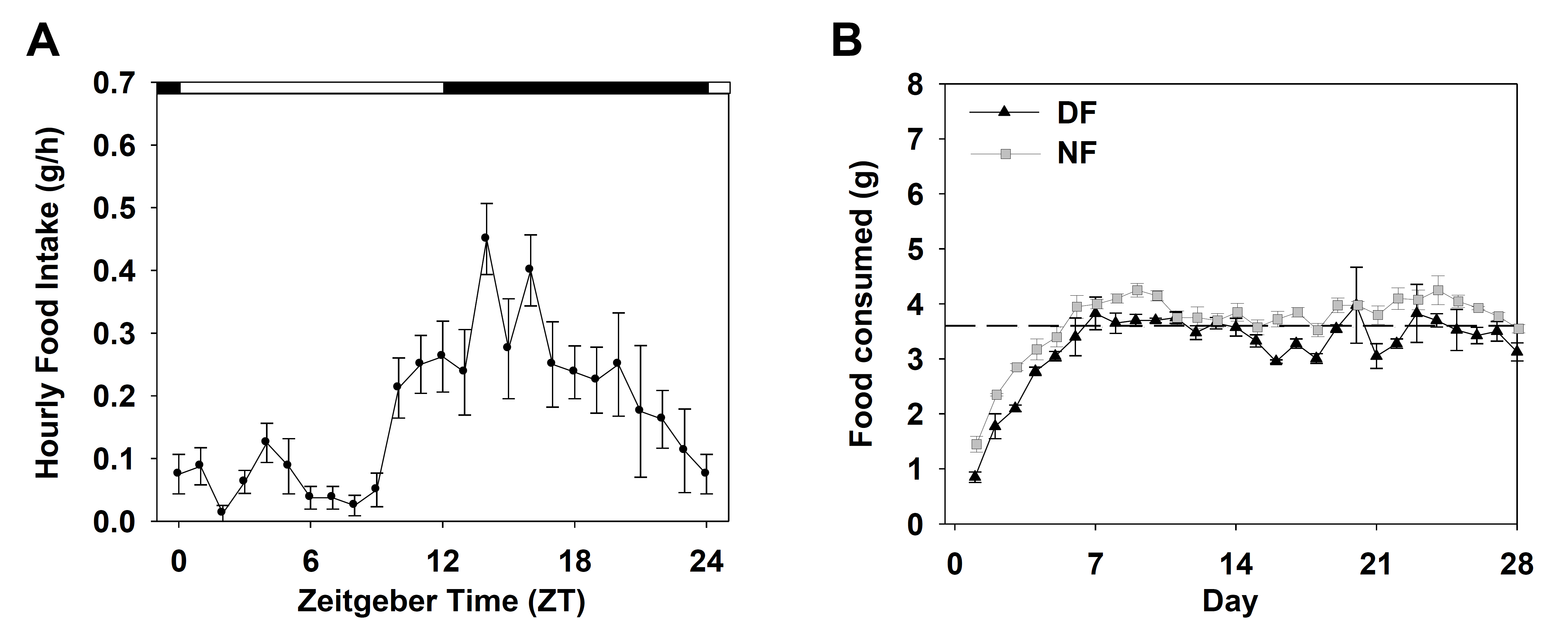

Supplement: Figure S1 — (A) Hourly food intakes in mice fed ad libitum. Adult C57BL/6J male mice (n = 16) were entrained to a 12∶12 LD photoperiodic cycle for a week in metabolic cages with food and water available all the time. On the 8th day, hourly food intake was determined throughout a day. (B) Time-course changes of daily food consumption in mice fed at either ZT06-11 (DF) or ZT18-23 (NF). Average daily food consumption in ad libitum fed mice is shown as a dashed line within the figure as a reference value. (TIF) [file pone.0044053.s001.tif]

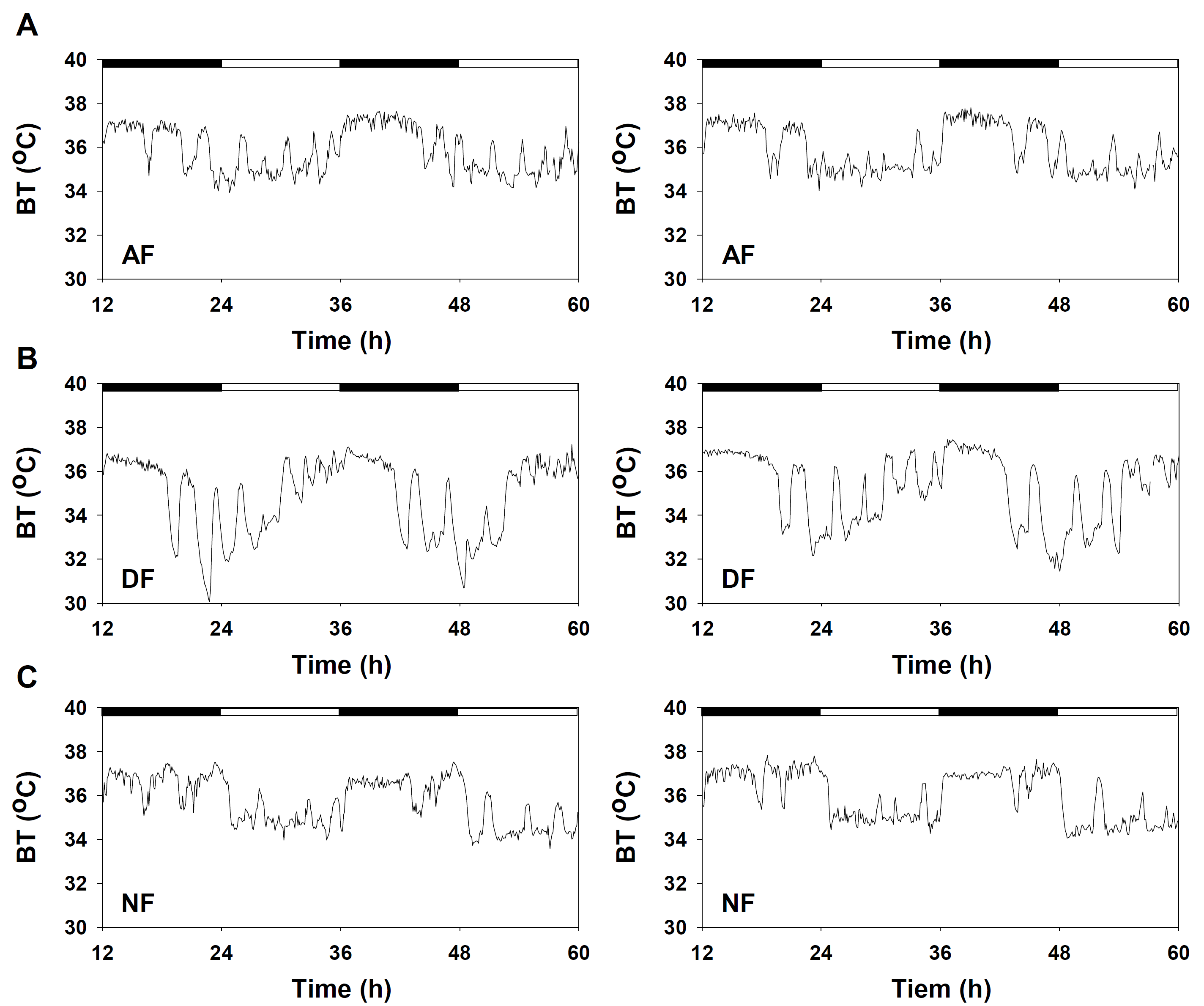

Supplement: Figure S2 — Representative 48 hour individual body temperature profiles in AF (A), DF (B) and NF (C) animals. Only in DF animals, rapid lowering of body temperature and recovering to normal ranges were repeated during the each fasting period. (TIF) [file pone.0044053.s002.tif]

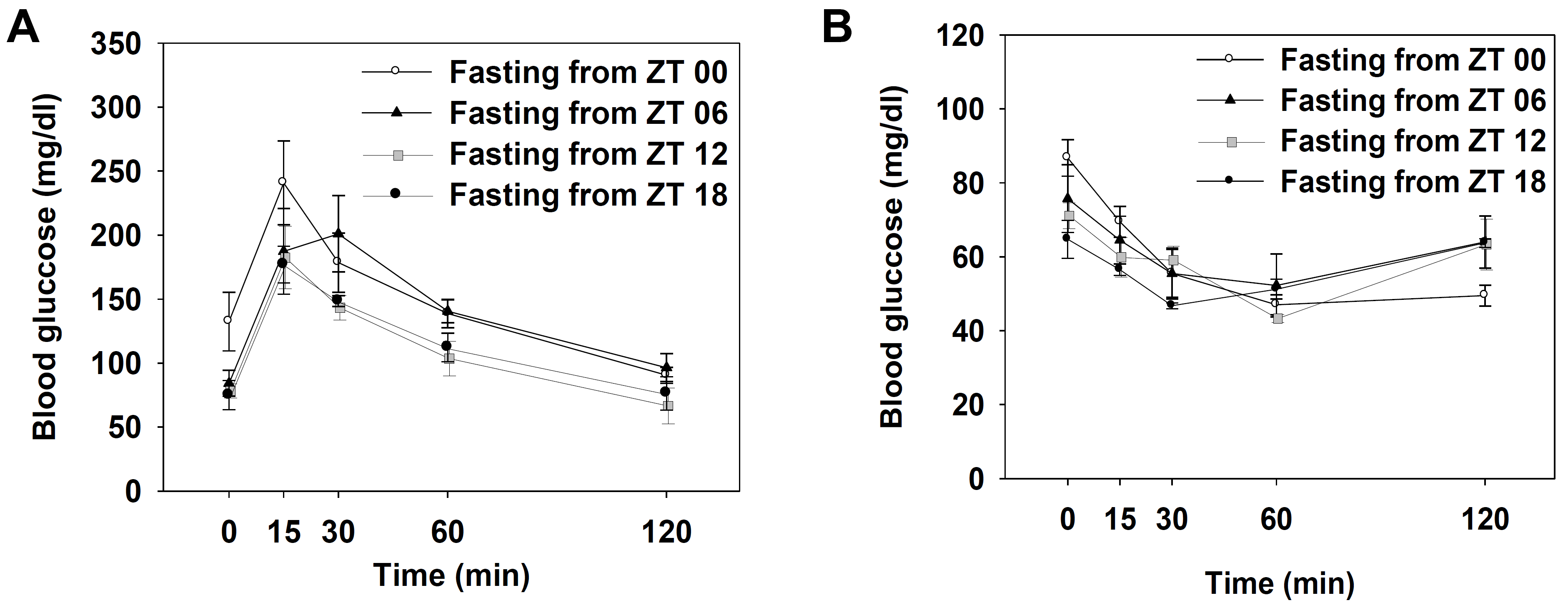

Supplement: Figure S3 — Time-of-day has little effects on OGTT and ITT results. Adult C57BL/6J male mice were fasted from the indicated zeitgeber time (ZT) for 16 h. Then, OGTT (A) and ITT (B) were performed as described in M&M of the main text. (TIF) [file pone.0044053.s003.tif]
